# Supplementary material for: Dual Detection of the Chytrid Fungi Batrachochytrium spp. with an Enhanced Environmental DNA Approach
Source: J Fungi (Basel). 2021 Mar 30;7(4):258. doi: 10.3390/jof7040258 (PMC8065926; doi:10.3390/jof7040258)
Supplement: Supplementary file 1 [file jof-07-00258-s001.pdf]

Supplementary Information: Dual detection of the chytrid fungi *Batrachochytrium* spp. with an enhanced environmental DNA approach focusing on simplicity and best storage.

Year	Article	Target	Environment	V(L)	FilterType (µm)	Pumping system	Storage	IPC	Kit Extraction	Purification/Antiinhibitor kit
2018	Hundermark & Takahashi	Amphibians	River	1	0.45 NC	Pump	Freeze		Blood and Tissue Kit Dneasy Qiagen (BTK)	
2018	Preissler & Watzal et al.	Amphibians	River	1	0.45NC	Pump	Freeze		Comparison	Comparison Qiagen vs Zymo
2018	Harper et al.	Review						Yes		
2018	Li et al.	Fish	Pond	0.3	Comparison	Pump/Syringe	Freeze		PowerWater DNA Mo Bio	SequalPrep (Invitrogen)
2018	Eiler et al.	Amphibians	Pond	0.5	0.45 Sx	Pump	Freeze		PowerSoil DNA	
2018	Wittwer et al.	Crayfish	Stream	up10	2.0 GF		Freeze			
2018	Fernandez et al.	Fish	Stream	1.5	0.2GF	Pump	Freeze		EZNA Tissue DNA Kit	
2017	Kamoroff & Goldberg	Bd	Lake	0.25	1.2PCTE	Pump	Ethanol		Dneasy BTK Qiagen	
2017	Mosher et al.	Bd	Laboratory	0.2	0.22 Sx	Syringe	Room	Yes	Gentra Puregene Tissue Kits	Zymo inhibitor removal kit
2017	Takahashi et al.	Amphibians	Stream	1	0.45 NC		Freeze	Yes	Dneasy BTK Qiagen	
2017	Trebitz et al.	Recommendations								
2017	Hinlo et al.	Comparison							PCI and CTAB	
2017	Agersnap et al.	Crayfish	Ponds	0.5-1.5	0.22 Sx	60mL Syringe	Freeze		CTAB	Qiagen QIAquick PCR purification
2017	Buxton et al.	Amphibians	Ponds	1	0.7 Glass	100 mL Syringe			Dneasy BTK Qiagen	
2017	Walker et al.	Amphibians							Dneasy BTK Qiagen	
2016	Spens et al.	Fish	Ponds	1	Comparison		Various			
2016	Goldberg et al.	Comparison								Zymo and Bovine Serum Albumine
2016	Civade et al.	Fish	Various	45		Pump	Buffer		Dneasy BTK Qiagen	MinElute PCR purification kit
2016	Lacoursiere-Roussel et al.	Fish	Aquaria	1	Comparison	Pump	Freeze			
2015	Valentini et al.	Review								
2015	Laramie et al.	Protocol		0.25		Various	Ethanol			
2017	DNeasy PowerWater Kit	Handbook	Various		0.22-0.45	Pump	Freeze		Dneasy PowerWater	
2015	Hall et al.	RV		0.25	0.2 NC	Pump	Ethanol	Yes	Qiashredder/Dneasy BTK	
2015	Eichmiller et al.	Fish	Tank	1	Comparison				FastDNA Spin Kit	Dilution 1/5
2015	Kolby et al.	Bd	Rainwater		0.22 Sx	Pump	Freeze		Qiagen ATL tissue lysis buffer/PrepMan	Dilution1/10
2014	Thomsen & Willerslev	Theoretical								
2014	Chestnut et al.	Bd	Ponds	0.35	0.22 Sx	60mL Syringe			Gentra Puregene Tissue Kit	Phosphate Buffered Saline
2014	Johnson & Brunner	RV		1	0.45 PVDF	Pump/Syringe	Freeze	Yes	Gentra Puregene Tissue Kit	
2014	Wimsatt et al.	Bd	Stream	0.32	0.8 Cellulose	Pump	Freeze		Fast DNA spin Kit	
2014	Rees et al.	Review							Several kits	
2013	Schmidt et al.	Bd	Ponds	0.6	0.2 Sx				Gentra Puregene Tissue Kit	Bovine Serum Albumine
2012	Hyman & Collins	Bd	Ponds	0.6	0.22 Sx	60mL Syringe	PBS/Freezer		Gentra Puregene Tissue Kit	Bovine Serum Albumine
2011	Strand et al.	Crayfish			3 PCTE		Freeze		CTAB	Bovine Serum Albumine
2011	Goldberg et al.	Amphibians	Stream	5.0-10	0.45NC	Pump	Ethanol		Dneasy BTK Qiagen+Qiagen Multiplex PCR	
2007	Walker et al.	Bd	Pond/Sediment	<1	0.45NC	50mL syringe	Freeze		MoBio Power Soil DNA	
2007	Kirshtein et al.	Bd	Pond/Sediment	<2.3	0.2 Sx	Pump	Freeze		Gentra Puregene Tissue/Ultraclean Soil DNA	MoBiocleanup/Generereleaser

Fig. S1. Review of manuscripts used to gather different methodologies and technics relevant for this research. *Batrachochytrium dendrobatidis* (Bd), Ranavirus (RV), Nitrate cellulose (NC), Sterivex (SX), Glass fibre (GF), Polycarbonate track-etched (PCTE), Polyvinylidene Fluoride (PVDF) and Internal Positive Control (IPC).

Table S2. Equipment needed per locality (1 filter or 20 swabs) for two people. Note that the following costs are not included: Gear disinfection, personal wages or salaries, perishable materials e.g. ladle, buckets, or any traps. It has to be highlighted that eDNA methodologies are more environmentally friendly as they use less plastic.

Gear Item (Prices in euros)	Lastra González et al. 2020 (eDNA)	Spitzen-van der Sluijs et al. 2020 (eDNA)	Swabs with QIAGEN	Swabs with PrepMan
Syringe	0.23	0.29	0	0
Filter	11.6	63	0	0
Silica	0.88	0	0.15	0.15
Falcon tube	1.08	0	0	0
Gloves (7.5cent/ud)	0.3	0.3	6	6
Bioline Meridian Bioscience Kit	3.5	0	0	0
Qiagen Blood and Tissue Kit/PrepMan	0	0	62.2	8.1
NucleoSpinSoil Macherey-Nagel Kit	0	4.3	0	0
Swabs (0,19 cents/ud) MWE	0	0	3.8	3.8
Internal Positive Controls (ThermoFisher)	1.5	0	0	0
Eppendorf for sampling (0,03/ud)	0	0	0.6	0.6
Ethanol 96%	0.06	0.97	0	0
Disposable plastic bags (individually placed amphibians)	0	0	1.3	1.3
Whirl-Pak bags	0	0.3	0	0
ATL Buffer Qiagen	0	0.32	0	0
Roche Master Mix	2.6	0	0	0
Environmental Master Mix	0	2.56	0	0
Standard Master Mix (0.36 eur/sample)	0	0	7.2	7.2
Total	21.75	72.04*	81.25	27.15

*According SPYGEN laboratories, it should be added a mandatory fieldwork training (80 euros/ person) and the costs of processing the samples (350 euros/ filter with two replicates). For that reason, other costs (e.g. conservation buffer) related to Spitzen-van der Sluijs et al. 2020 eDNA approach are impossible to calculate precisely. In any case, it is a conservative estimate.

Table S3. Ct values corresponding to the storage methods experiment where *Batrachochytrium salamandrivorans* (*Bsal*) primers were tested from two different articles. Internal Positive Control (IPC) just included in the first analyses to discard PCR inhibition. Control filter (CF), Silica gel filter (Sil), Longmire's buffer (LB), Ethanol (EtOH). Blank space means not qPCR positive detection.

Storage method	Primer's Ct values from Blooi et al. 2013	Storage method	Primer's Ct values from Spitzen-van der Sluijs et al. 2020
CF1	39.4	CF1	39.93
CF1		CF1	
CF1	IPC	CF1	39.31
CF2		CF2	
CF2		CF2	
CF2	IPC	CF2	
CF3	36.68	CF3	38.44
CF3	37.93	CF3	36.1
CF3	IPC	CF3	35.97
LB1	39.5	LB1	36.47
LB1	35.67	LB1	34.8
LB1	IPC	LB1	
LB2	35.52	LB2	36.07
LB2	35.82	LB2	36.69
LB2	IPC	LB2	36.58
LB3	35.62	LB3	36.83
LB3	35.54	LB3	38.3
LB3	IPC	LB3	36.93
Sil1	33.07	Sil1	34.71
Sil1	33.98	Sil1	34.62
Sil1	IPC	Sil1	34,56
Sil2	36.58	Sil2	36.64
Sil2	37.17	Sil2	36.83
Sil2	IPC	Sil2	33.47
Sil3	32.77	Sil3	33.94
Sil3	32.66	Sil3	33.97
Sil3	IPC	Sil3	34.32
EtOH1	34.77	EtOH1	36.69
EtOH1	34.63	EtOH1	35.64
EtOH1	IPC	EtOH1	36.11
EtOH2	32.53	EtOH2	34.03
EtOH2	32.95	EtOH2	34.31
EtOH2	IPC	EtOH2	33.81
EtOH3	32.23	EtOH3	33.74
EtOH3	31.66	EtOH3	33.75
EtOH3	IPC	EtOH3	33.22

Table S4. Comparison between our results and an independent university as control from eDNA filters.

Locality	University of Veterinary and Pharmaceutical Sciences Brno*	Trier University*
Ampuero	10.81	130
Teverga 1	96.55	978
Teverga 2	22.35	650
Ruente 1	Negative sample	84
Suances	96.05	528
Ponga	24.2	227
Cieza	33.3	1149

*Numbers are DNA copies

Table S5. Volumes, pore size filter, storage method and Ct value of each filter. Number of filters (N). Distances within Ruente and Teverga localities are at least 5 km.

Locality (N)	Volume filtered (mL)	Pore size (μm)	eDNA Storage Method	Ct value (Mean of wells)
Ampuero	400	0.22	Longmire's Buffer#	36.92
Teverga 1	1000	0.45	Silica gel#	33.62
Teverga 2	650	0.45	Silica gel	33.63
Teverga 3* (2)	74/88	0.45/0.45	Longmire's Buffer	36.75/36.70
Ruente 1 (2)	1000/1000	0.22/0.45	Silica gel	36.76/Negative
Ruente 2	325	0.22	Longmire's Buffer	37.76
Suances (2)	123/158	0.22/0.22	Longmire's Buffer /Silica gel	35.85 /36.38
Ponga* (2)	1000/409	0.45/0.22	Longmire's Buffer	39.88/35.71
Cieza	138	0.45	Longmire's Buffer	35.24

*Two different water habitats but close to each other. Numbers in brackets are number of filters.
#Longmire's buffer: (100 mM Tris, 100 mM EDTA, 10 mM NaCl, 0.5 % SDS, 0.2 % sodium azide) and Silica gel (orange indicator, 2-5 mm, P-lab, Prague, Czech Republic)
